# The protective role of religiosity against problem gambling: findings from a five-year prospective study

**DOI:** 10.1186/s12888-017-1518-5

**Published:** 2017-11-06

**Authors:** Seema Mutti-Packer, David C. Hodgins, Robert J. Williams, Barna Konkolÿ Thege

**Affiliations:** 1 Department of Psychology, University of Calgary, 2500 University Drive NW, Calgary, AB T2N 1N4 Canada; 2 Faculty of Health Sciences, University of Lethbridge, Lethbridge, AB Canada; 3 Research and Academics Division, Waypoint Centre for Mental Health Care, Penetanguishene, ON Canada; 4 Department of Psychiatry, University of Toronto, Toronto, ON Canada

**Keywords:** Trajectory of gambling, Adults, Problem gambling, Religion, Religious affiliation, Longitudinal, Latent growth curve modeling

## Abstract

**Background:**

Little research has examined the potential protective influence of religiosity against problem gambling; a common addictive behavior, and one with a host of associated negative health and social outcomes. The aims of this study were to examine (1) the potential longitudinal association between religiosity and problem gambling among adults and (2) the potential moderating role of gender on this association.

**Methods:**

Data were from five waves of the Quinte Longitudinal Study (QLS), between 2006 and 2010. Participants were Canadian adults from Belleville, Ontario, Canada (*n* = 4121). A multiple group (based on gender) latent growth curve analysis was conducted to examine the overall trajectory of problem gambling severity. Two models were tested; the first examined the influence of past-year religious service attendance, and the second examined an overall measure of personal religiosity on the trajectory of problem gambling. The Problem and Pathological Gambling Measure (PPGM) was used as a continuous measure. The Rohrbaugh-Jessor Religiosity Scale (RJRS) was used to assess past-year frequency of religious service attendance and personal religiosity. Religious affiliation (Protestant, Catholic, Atheist/Agnostic, Other, Prefer not to say) was also included in the models.

**Results:**

At baseline, higher frequency of past-year religious service attendance (males: β= −0.54, females: β= −0.68, *p < 0.001* for both) and greater overall personal religiosity (males: β= −0.17, females: β= −0.13, *p < 0.001* for both) were associated with lower PPGM scores. The moderating effect of gender indicated that the influence of past-year religious service attendance was greater among females (χ^2^diff_(44)_ *=* 336.8, *p < 0.001*); however, the effect of overall religiosity was greater among males (χ^2^diff_(36)_ *=* 213.4, *p < 0.001*). Findings were mixed with respect to religious affiliation. No measures of religiosity or religious affiliation were associated with the overall decline in problem gambling severity.

**Conclusions:**

These findings suggest that religiosity may act as a static protective factor against problem gambling severity but may play a less significant role in predicting change in problem gambling severity over time.

**Electronic supplementary material:**

The online version of this article (10.1186/s12888-017-1518-5) contains supplementary material, which is available to authorized users.

## Background

A large body of evidence indicates that religion and religious involvement are associated with positive mental health and physical health outcomes, including decreased rates of depression, suicide, and coronary heart disease [1]. There is also research highlighting the protective influence of religiosity against addictive behaviors such as smoking, alcohol and drug use [1, 2]. However, there has been relatively little research which examines the potential protective influence of religiosity against excessive gambling, a common addictive behavior, and one with a host of associated negative outcomes including criminal behavior [3], psychiatric comorbidities [4], and financial issues [5]. The limited amount of research in the field has indicated that religious involvement is inversely related to ever-gambling [6, 7], past-year gambling [8], gambling frequency, and the amount of money gambled [9–11].

It is important to note that religiosity is multi-dimensional in nature, and has both a public and private component. For example, attending religious services can be considered a public display or community aspect of religiousness, whereas the extent to which someone internalizes their faith and uses it to guide their life and decision-making can be considered the private component. In addition to public and private components of religiosity, religious affiliation represents another dimension of religiosity that is important to understand in the context of gambling. Religious traditions tend to have diverse ethical codes of conduct with regards to gambling. For example, although gambling is not explicitly prohibited in the Bible, conservative and mainline Protestant groups interpret its teachings as such. Within the Islamic tradition, gambling is explicitly prohibited according to the Quran. On the other hand, there are religious traditions that do not reject gambling behavior categorically, such as the Catholic and Jewish traditions. Overall, religious affiliation and its codes of conduct have the potential to shape cultural norms, beliefs, and ultimately behavior.

One major methodological limitation of studies examining the influence of various dimensions of religiosity on gambling is that most are cross-sectional, and unable to examine these associations over time. To our knowledge, only one study has tested the longitudinal association between religiosity and gambling [6]. This study used data from the US National Longitudinal Study of Adolescent to Adult Health, and found that religiousness during adolescence predicted aspects of gambling behavior in young adulthood. Additional longitudinal research is needed to assess the potential protective influence of religiosity on subsequent problem gambling behaviors in other jurisdictions and among different age cohorts.

Moreover, despite evidence showing that men and women differ in terms of the occurrence, prevalence, correlates, and consequences of their addictive behaviors [12, 13], the role of gender has not yet been explicitly examined in studies related to gambling and religiosity. It has been hypothesized that religiosity is influenced by underlying personality traits, namely risk aversion [14, 15]. This line of thinking posits that those who are more risk-averse, are also more likely to be religious. This notion has been used to explain consistent gender differences across various dimensions of religiosity indicating that women tend to be more religious overall [14, 15]. However, a recent study found no evidence supporting the idea that risk-aversion explains the association between religion and gambling [6].

The primary goal of the current study was to examine the potential protective influence of religious service attendance and overall religiosity on problem gambling severity over time, among a sample of Canadian adults. Specific aims were to: 1) examine the trajectory of problem gambling severity, and 2) examine the potential influence of religious service attendance and personal religiosity on problem gambling severity both at baseline and over time, and 3) examine the potential moderating role of gender on these associations.

## Methods

### Sample and procedure

Data were from the Quinte Longitudinal Study (QLS) [16], the primary aim of which was to help develop an etiological model of problem gambling. Ethical approval was provided by the Human Subject Research Committee at the University of Lethbridge. A cohort of 4121 adults from Ontario, Canada were assessed yearly over a five-year period (2006 to 2011).

Recruitment was conducted via random digit telephone dialing from a pool of numbers with area codes and prefixes estimated to be within 70 km of the city of Belleville in Ontario, Canada. The response rate in the QLS was 21.3%, a similar value to those obtained in research with a similar focus from the same country e.g., [17]. The retention rate for the QLS was 93.9%, an exceptionally high value in large scale longitudinal research of this nature. Two samples were recruited: a ‘general population’ sample (*n = 3065*) and an ‘at risk’ sample for problem gambling (*n = 1056*). The purpose of recruiting the ‘at risk’ subsample was to ensure that there were a sufficient number of people in the cohort who became problem gamblers during the course of the study. For a respondent to be considered ‘at-risk’, they had to indicate one or more of the following: (1) spending $10 or more per month on lottery, instant win tickets, bingo, casino table games, or games of skill against other people; (2) playing either slot machines or betting on horse racing in the past year; (3) an intention to gamble at a new slots-at-racetrack facility that was scheduled to be built sometime in the next few years in the area.

If the person agreed to participate in the survey, they were sent an email with a link to the online questionnaire or booked into a time slot at the program office where they completed the survey on a computer on site. Informed written consent was obtained from all participants prior to completing the online survey, each year. A total of 69.5% opted to complete the survey online for the first assessment, with this proportion steadily increasing to 90.0% for the final assessment. A small percentage of people completed a paper and pencil version of the survey because of their unfamiliarity with computers (1.2% to 1.9% depending on the survey year). Further methodological details can be found elsewhere [18].

The focus of the QLS was to examine changes in gambling over time, thus it was not necessary that the sample be representative of the Ontario or Canadian population, only that it contained a diverse range of gamblers. Nonetheless, the demographic profile of the sample is similar to Canadian adults (15+) as established by the 2006 Canadian Census, with the exception that the present sample tends to include slightly fewer people aged 18 to 24, seniors 65 and older, single people, and has a somewhat higher level of educational attainment. Characteristics of the study sample are described in Table 1.

**Table 1:** Baseline sample characteristics

	Overall (*n = 4121)*	Males (*n = 1867*)	Females (*n = 2253*)
Age [M (SD)]	46.1 (14.1)	47.1 (14.7)	45.2 (13.6)
Overall religiosity^a^ [M (SD)]	12.1 (7.0)	10.8 (7.1)	13.1 (6.6)
Past year religious service attendance^b^ [N (%)]			
Missing	162 (3.9)	103 (5.5)	59 (2.6)
Not at all	1654 (41.8)	758 (43.0)	896 (40.8)
Low	1307 (33.1)	568 (32.2)	739 (33.7)
Moderate	462 (11.7)	189 (10.7)	273 (12.4)
High	536 (13.5)	249 (14.1)	287 (13.1)
Religious affiliation [N (%)]			
Missing	1 (0.0)	–	1 (0.0)
Catholic	860 (29.0)	391 (22.3)	469 (22.5)
Protestant	2269 (55.1)	977 (55.8)	1292 (62.1)
Agnostic/atheist	331 (8.0)	195 (11.1)	136 (6.5)
Other^c^	372 (9.0)	189 (10.8)	183 (8.8)
Prefer not to say	288 (7.0)	115 (6.2)	173 (7.7)
Education [N (%)]			
Less than high school	462 (11.2)	236 (12.6)	226 (10.0)
High school	823 (20.0)	355 (19.0)	468 (20.8)
Some post-secondary or technical school	1106 (26.8)	561 (30.0)	545 (24.2)
Completed college/university or more	1730 (42.0)	715 (38.3)	1015 (45.0)
Annual household income^d^ [N (%)]			
$0 to $39,999.00	1402 (34.0)	557 (29.8)	845 (37.5)
$40,000.00 to $79,999.00	1716 (41.6)	793 (42.5)	923 (40.9)
$80,000.00 or more	1003 (24.3)	517 (27.7)	486 (21.6)
Marital status [N (%)]			
Never married	491 (11.9)	236 (12.6)	255 (11.3)
Married or common-law	2944 (71.4)	1387 (74.3)	1557 (69.1)
Divorced/separated/widowed	686 (16.6)	244 (13.1)	442 (19.6)

^a^Items 2–8 of the Rohrbaugh Jessor Religiosity Scale

^b^Low = categories “once” and “2 to 5 times” in the past year; Moderate = categories “6 to 10 times” and “once or twice a month”; High: categories “once a week” and “more than once a week”

^c^Other’ religious affiliation includes Muslim, Jewish, Buddhist, Hindu, Sikh, and other affiliations

^d^‘Unsure’ responses (*n* = 129) were replaced by imputed values: if income stable in next 2 data waves then replaced with that value; elsewhere replaced with the mean of values from all other survey waves

### Measures

Severity of gambling problems was assessed by the Problem and Pathological Gambling Measure (PPGM) [19, 20]. The PPGM is a 14-item item instrument that provides both a continuous measure of problem gambling severity, as well as a categorical risk assessment that classifies people into the following categories: non-gambler; recreational gambler; at risk gambler; problem or pathological gambler. The PPGM has good internal consistency (Cronbach α =0.76 to 0.81, depending on the dataset) and 1 month test-re-test reliability (*r* = 0.78), as well as excellent correspondence to clinical assessment [20].

Religiosity was measured by the Rohrbaugh–Jessor Religiosity Scale (RJRS) [21]. This scale is an eight-item instrument assessing ritual, consequential, ideological, and experiential religiosity. Internal consistency of the scale was excellent (Cronbach α = .90). The first item of the scale, referring to past-year frequency of religious service attendance was examined separately in order to examine the community aspect of religious involvement, similar to previous research [6]. Response options for the measure of past-year religious service attendance were: 0 = Not at all; 1 = Once; 2 = Two to five times; 3 = Six to ten times; 4 = Once or twice a month; 5 = Once a week; 6 = More than once a week. Following Uecker and Stokes [6], responses were categorized as: ‘Not at all’, ‘Low’ = “Once” and “Two to five times”; ‘Moderate’ = “Six to ten times” and “Once or twice a month”; ‘High’ = “Once a week” and “More than once a week”.

The seven remaining items of the RJRS assessed the magnitude of religious belief in a person’s life, and each question had four or five response options indicating a range of agreement or importance. Specifically, five questions had five response options, and two questions had four response options. For example, response options for the following question: “When you have a serious personal problem, how often do you take religious advice or teaching into consideration?”, were: “almost always, usually, sometimes, and never” (scored 3, 2, 1, and 0, respectively). The responses from these seven items were summed together, with a range of scores falling between 0 and 26; higher scores indicated greater belief.

To assess one’s religious affiliation, respondents were asked ‘What is your religious affiliation?’ Response options were: Catholic, Protestant (e.g., Anglican, Baptist, Lutheran, United, Presbyterian), Atheist, Agnostic, Other (e.g., Muslim, Jewish, Buddhist, Hindu, Sikh), or Prefer not to say.

### Statistical analyses

Mplus version 6.0 was used for all analyses. Following a stepwise-procedure [22], the first step was to fit a baseline or unconditional latent growth curve model to examine the overall trajectory of problem gambling severity. Next, the variables of interest were added to the model. To examine the potential protective effects of religiosity on problem gambling severity over time, two models were tested. The first model examined the frequency of past-year religious service attendance. The second model included an overall measure of religiosity. Both models included religious affiliation, and adjusted for age, education, household income, and marital status. This analytic approach employed a full-information maximum likelihood (FIML) estimator.

Latent growth curve models include two latent factors, the intercept and slope, as well as repeated measures of the observed outcome of interest over time. The slope factor loadings for the unconditional linear model were fixed at 0, 1, 2, 3, and 4, defining the start of the study as the intercept. To examine the potential moderating influence of gender, a multiple-group approach was used, whereby each level of the moderator was specified as a group (male and female). The multiple groups approach estimates growth curves based on the grouping of interest within a single analysis. Differential effects were examined by fixing regression co-efficients to be equal across groups (also known as equality constraints) and comparing the constrained model to the unconstrained model. If the chi-square statistic of the constrained model had increased significantly, the unconstrained model fit the data better than the constrained model, indicating a significant moderating effect of the grouping variable (gender in this case).

The following indices were used to examine model fit: comparative fit index (CFI), Tucker-Lewis index (TLI), root mean square error of approximation (RMSEA), and standardized root mean square residual (SRMR). Adequate fit was indicated by CFI and TLI >0.90, RMSEA <0.08, and SRMR <0.10. Good fit was indicated by CFI and TLI >0.95, RMSEA <0.06, and SRMR <0.08 [23]. Important to note is that chi-square test of model fit is likely to be significant when the sample size is large, which is the case for the current study. Thus, model fit was based primarily on the CFI, TLI, RMSEA, and SRMR.

## Results

### Unconditional latent growth curve model

An overall decreasing trend that appeared linear was observed in mean scores based on the PPGM assessment across the five assessments (Table 2). The linear growth curve model fit the data well [χ^2^_(19)_ = 42.7, *p* = 0.001, CFI = 0.99, TLI = 0.99, RMSEA = 0.03, SRMR = 0.02]. The mean latent variables representing the intercept and slope indicated similar baseline levels of problem gambling severity for males and females, as well as the same decreasing slope over time (Table 3). For both males and females, there was also significant variance in baseline levels of problem gambling severity, indicating that not everyone started at the same level of problem gambling severity. The significant variance in the slope indicated variability in individual trajectories over time. The significant negative correlations between the intercept and slope indicate that higher levels of problem gambling severity at baseline were associated with steeper declines over time. To test the moderating effect of gender, the constrained [χ^2^_(21)_ = 47.02] and unconstrained [χ^2^_(19)_ = 42.72] models were compared, and the results [χ^2^diff_(2)_ = 4.3, *p = 0.12*] indicated no moderating effect of gender.

**Table 2:** Gambling severity categories and mean scores (SD) based on the Problem and Pathological Gambling Measure (PPGM) across the five data waves

	Time 1	Time 2	Time 3	Time 4	Time 5
Overall					
Sample size at given data wave	4120	3939	3901	3829	3799
Non-gambler [N (%)]	309 (7.5)	298 (7.6)	363 (9.3)	423 (11.0)	406 (10.7)
Recreational gambler [N (%)]	3111 (75.5)	3092 (78.5)	3034 (77.8)	2978 (77.8)	2951 (77.7)
At-risk gambler [N (%)]	564 (13.7)	436 (11.1)	401 (10.3)	324 (8.5)	365 (9.6)
Problem/pathological gambler [N (%)]	136 (3.3)	113 (2.9)	103 (2.6)	104 (2.7)	77 (2.0)
PPGM score^a^ [M (SD)]	1.13 (0.57)	1.09 (0.54)	1.06 (0.54)	1.03 (0.55)	1.03 (0.53)
Males					
Sample size at given data wave	1867	1770	1742	1713	1690
Non-gambler [N (%)]	135 (7.2)	127 (7.2)	165 (9.5)	194 (11.3)	186 (11.0)
Recreational gambler [N (%)]	1385 (74.2)	1376 (77.7)	1321 (75.8)	1322 (77.2)	1278 (75.6)
At-risk gambler [N (%)]	281 (15.1)	222 (12.5)	215 (12.3)	149 (8.7)	187 (11.1)
Problem/pathological gambler [N (%)]	65 (3.5)	45 (2.5)	41 (2.4)	48 (2.8)	39 (2.3)
PPGM score^a^ [M (SD)]	1.15 (0.58)	1.10 (0.54)	1.08 (0.55)	1.03 (0.56)	1.05 (0.56)
Females					
Sample size at given data wave	2253	2169	2159	2116	2109
Non-gambler [N (%)]	174 (7.7)	171 (7.9)	198 (9.2)	229 (10.8)	220 (10.4)
Recreational gambler [N (%)]	1725 (76.5)	1716 (79.1)	1713 (79.3)	1656 (78.3)	1673 (79.3)
At-risk gambler [N (%)]	283 (12.6)	214 (9.9)	186 (8.6)	175 (8.3)	178 (8.4)
Problem/pathological gambler [N (%)]	71 (3.1)	68 (3.1)	62 (2.9)	56 (2.6)	38 (1.8)
PPGM score^a^ [M (SD)]	1.11 (0.56)	1.08 (0.54)	1.05 (0.54)	1.03 (0.54)	1.02 (0.51)

^a^ Missing values imputed: Non-gamblers coded to 0; infrequent/low spend gamblers coded to 1. Gambling categories are listed for descriptive purposes only; continuous PPGM scores were used for analyses

**Table 3:** Unconditional latent growth curve model of problem gambling severity, by gender (n = 4120)

	Males (n = 1867)		Females (n = 2253)	
	estimate	p	estimate	p
Means/intercept	1.14	<0.001	1.10	<0.001
Means/slope	−0.03	<0.001	−0.03	<0.001
Variances/intercept	0.20	<0.001	0.20	<0.001
Variances/slope	0.005	<0.001	0.003	<0.001
Within-process correlation (intercept < −>slope)	−0.32	<0.001	−0.44	<0.001

Unstandardized estimates were used for means and variances; standardized estimates were used for correlations

### Conditional latent growth curve model: The influence of past-year frequency of religious service attendance

The conditional latent growth curve model estimating the effect of past-year religious service attendance showed good fit [χ^2^_(37)_ *=* 55.5, *p = 0.03*, CFI = 0.99, TLI = 0.99, RMSEA = 0.02, SRMR = 0.01]. Among both males and females, those with high religious service attendance reported significantly lower levels of problem gambling severity at baseline (β = −0.62, *p < 0.001*, and β = −0.65, *p < 0.001*, respectively) compared to those who did not attend religious services at all in the past year. For females, even those with moderate past year attendance reported significantly lower levels of problem gambling severity at baseline (β = −0.16, *p = 0.04*) compared to those who did not attend at all*.* Furthermore, past-year religious service attendance influenced the slope of problem gambling severity for females, meaning that those with high past-year attendance, experienced a slower decline in problem gambling severity (β = 0.32, *p = 0.03*). In terms of the moderating effect of gender, a significant difference between the constrained and unconstrained model [χ^2^diff_(13)_ = 192.9, *p < 0.001*] was observed indicating a moderating effect of gender on frequency of service attendance, such that the effect of past-year religious service attendance on problem gambling severity was greater among females.

The model with covariates also indicated good fit [χ^2^_(91)_ = 115.5, *p = 0.04,* CFI = 0.99, TLI = 0.99, RMSEA = 0.01, SRMR = 0.01]. Table 4 presents the results from this model. All significant associations between past-year service attendance and problem gambling severity, as well as the moderating effect of gender [χ^2^diff_(44)_ = 336.8, *p < 0.001*], were retained after including covariates in the model, with the exception of the influence of high past-year religious service attendance on the slope of problem gambling severity for females, which lost significance.

**Table 4:** The influence of frequency of religious service attendance on the intercept and slope of problem gambling severity, by gender (*n = 3959*)

	Males (*n* = 1764)				Females (*n* = 2195)			
	Intercept		Slope		Intercept		Slope	
	Effect	p	Effect	p	Effect	p	Effect	p
Frequency of service attendance^a^								
Not at all (ref.)	–	–	–	–	–	–	–	–
Low	0.10	0.11	−0.16	0.15	0.03	0.57	0.04	0.68
Moderate	−0.07	0.48	−0.005	0.98	−0.16	0.04	0.06	0.68
High	−0.54	<0.001	−0.25	0.10	−0.68	<0.001	0.24	0.10
Religious affiliation^b^								
Atheist/Agnostic (ref.)	–	–	–	–	–	–	–	–
Catholic	0.22	0.11	0.01	0.96	0.30	0.03	−0.09	0.74
Protestant	0.06	0.66	−0.08	0.73	0.18	0.17	−0.01	0.98
Other	−0.10	0.50	0.02	0.93	−0.13	0.41	−0.01	0.97
Prefer not to say	0.21	0.19	−0.38	0.19	0.16	0.29	−0.13	0.66
Age	−0.004	0.04	0.01	0.01	0.003	0.17	0.006	0.10
Education	−0.12	<0.001	0.02	0.67	−0.14	<0.001	0.05	0.25
Household income	0.07	0.08	−0.04	0.60	0.02	0.465	0.10	0.12
Marital status								
Never married (ref.)	–	–	–	–	–	–	–	–
Married or common law	−0.34	<0.001	0.36	0.03	−0.20	0.02	0.42	0.01
Divorced or separated or widowed	−0.11	0.33	0.06	0.78	−0.19	0.05	0.45	0.01

Standardized estimates used for regression coefficients

^a^ Low = categories “once” and “2 to 5 times” in the past year; Moderate = categories “6 to 10 times” and “once or twice a month”; High: categories “once a week” and “more than once a week”

^b^ See Additional file 1: Table S1 for contrasts between all levels of religious affiliation

Differences also emerged with respect to religious affiliation (Table 4 and Additional file 1: Table S1). Catholic males had significantly higher levels of problem gambling severity at baseline compared to Protestant males (β = 0.16, *p = 0.02*), and males who reported other religious affiliations (β = 0.32, *p = 0.002*). Catholic females had significantly higher levels of problem gambling severity at baseline compared to Protestant females (β = 0.12, *p = 0.05),* those who reported Other religious affiliations (β = 0.43, *p < 0.001*), as well as those who were Atheist/Agnostic (β = 0.30, *p = 0.03*). Protestant females had greater levels of problem gambling severity at baseline compared to those with Other religious affiliations (β =0.31, *p = 0.001*). In addition, males and females who preferred not to state their religious affiliation reported greater levels of problem gambling severity at baseline compared to those who reported other religious affiliations (β =0.31, *p = 0.02* and β =0.29, *p = 0.02*). Religious affiliation did not influence the slope of problem gambling severity for males or females.

### Conditional latent growth curve model: The influence of overall religiosity on problem gambling severity

The conditional latent growth curve model estimating the effect of past-year religiosity showed good fit [χ^2^_(25)_ = 47.7, *p = 0.004,* CFI = 0.99, TLI = 0.99, RMSEA = 0.02, SRMR = 0.02]. For both males and females, overall religiosity was negatively associated with baseline levels of problem gambling severity, meaning that those with higher levels of religiosity reported lower levels of problem gambling severity at baseline (β = −0.17, *p < 0.001,* and β = −0.09, *p < 0.001*, respectively). The moderating effect of gender was significant [χ^2^diff_(5)_ = 45.9, *p < 0.001*], indicating that the effect of religiosity on baseline levels of problem gambling severity was greater among males.

The model with covariates fit well [χ^2^_(79)_ = 113.9, *p = 0.01,* CFI = 0.99, TLI = 0.99, RMSEA = 0.01, SRMR = 0.01]. Table 5 presents the results from this model. All significant associations between religiosity and problem gambling severity, as well as the moderating effect of gender [χ^2^diff_(36)_ = 213.4, *p < 0.001*], were retained after including covariates in the model. Differences emerged with respect to religious affiliation (Table 5 and Additional file 2: Table S2). Catholic males had significantly higher levels of problem gambling severity at baseline compared to all other religious affiliations (Protestant: β = 0.14, *p = 0.04*; Atheist/Agnostic: β = 0.28, *p = 0.01*; Other: β = 0.33, *p = 0.001*), except for those who preferred to not state their religious affiliation. Protestant males reported greater levels of problem gambling severity compared to those with other religious affiliations (β = 0.19, *p = 0.04*). In addition, males and females who preferred not to state their religious affiliation, reported higher levels of problem gambling severity at baseline compared to those who reported other religious affiliations (β =0.28, *p = 0.04* and β =0.33, *p = 0.01*, respectively).

**Table 5:** The influence of overall religiosity on the intercept and slope of problem gambling severity, by gender *(n = 4120)*

	Males (n = 1867)				Females (n = 2253)			
	Intercept		Slope		Intercept		Slope	
	Effect	p	Effect	p	Effect	p	Effect	p
Religiosity total score	−0.17	<0.001	−0.002	0.97	−0.13	<0.001	−0.004	0.93
Religious affiliation^a^								
Atheist/Agnostic (ref.)	–	–	–	–	–	–	–	–
Catholic	0.28	0.01	−0.15	0.45	0.49	<0.001	−0.15	0.51
Protestant	0.14	0.15	−0.22	0.21	0.41	<0.001	−0.09	0.67
Other	−0.05	0.71	−0.11	0.63	0.05	0.71	−0.09	0.71
Prefer not to say	0.23	0.10	−0.46	0.07	0.38	0.005	−0.25	0.33
Age	−0.01	0.004	0.01	0.01	0.001	0.51	0.01	0.10
Education	−0.15	<0.001	0.02	0.63	−0.14	<0.001	0.06	0.22
Household income	0.07	0.06	−0.05	0.43	0.01	0.75	0.11	0.11
Marital status								
Never married (ref.)	–	–	–	–	–	–	–	–
Married or common law	−0.33	<0.001	0.45	0.005	−0.19	0.03	0.43	0.01
Divorced or separated or widowed	−0.05	0.66	0.15	0.46	−0.15	0.14	0.48	0.01

^a^ See Additional file 2: Table S2 for contrasts between all levels of religious affiliation

Catholic and Protestant females showed the same pattern of results. Both religious groups reported significantly higher levels of problem gambling severity at baseline compared to those who were atheist/agnostic, as well as those who reported other religious affiliations (Catholic females: β = 0.49, *p < 0.001* and β = 0.44, *p < 0.001*, respectively; Protestant females: β = 0.41, *p < 0.001* and β = 0.36, *p < 0.001*, respectively). In addition, females preferred not to state their religious affiliation reported greater levels of problem gambling severity at baseline compared to those who reported they were Atheist/Agnostic (β = 0.38, *p = 0.005*). Similar to overall religiosity, religious affiliation did not influence the slope of problem gambling severity either for males or females.

## Discussion

The findings from the current study are consistent with previous longitudinal research demonstrating the transient nature of problem gambling [24]. In addition, there was a negative correlation between baseline levels of problem gambling severity and its rate of change: higher levels of problem gambling severity at baseline were associated with steeper declines over time. These findings are also similar to those from a recent study examining the latent growth trajectory of problem gambling severity using data from the Manitoba Longitudinal Study of Young Adults [25]. This naturally occurring, decreasing pattern has also been observed in trajectories of other behavioral addictions. For example, one study using data from the QLS examined trajectories of various normatively counter-indicated habits including excessive eating, sexual behavior, shopping, online chatting, eating, and video gaming, and found that the excessive behavior was highest at the initial assessment, followed by a decreasing trend [26]. Together, this set of findings suggests that problem gambling is episodic in nature, and at the sub-clinical level, may resolve naturally over time. Additionally, given that the QLS included non-clinical samples, it is possible that the survey itself may have acted as an intervention; cueing respondents to think about their behavior and to make changes over time.

The results of the present study are also in line with previous findings indicating that religious service attendance may be a more robust protective factor against gambling than other aspects of religiosity [27–29]. Specifically, we found that respondents who attended religious services weekly or more reported lower levels of problem gambling severity. Similarly, Uecker and Stokes [6] found that adolescents who attended religious services weekly or more had lower odds of having ever gambled. In terms of longitudinal associations in the current study, only weekly or more frequent past-year religious service attendance had a significant association with the slope of problem gambling severity. This association was significant only for females, and did not occur in the expected direction; those who attended religious services weekly or more experienced a slower decline in problem gambling severity over time. It is possible that the slower rate of decline was due to the fact that respondents who attended religious services weekly or more had lower levels of problem gambling severity to begin with. However, this association lost significance once covariates were added to the model, indicating that religious service attendance did not independently influence problem gambling severity over time. The moderating effect of gender indicated that the influence of past-year religious service attendance was greater among females; however, the effect of overall religiosity was greater among males. However, it is important to note that strong conclusions cannot be drawn without also examining the role of selection factors, such as personality factors, that might make it more likely that individuals attend religious services, and less likely to develop gambling problems. For example, conscientiousness has been found to be negatively associated with problem gambling [30]. Within the construct of conscientiousness, the facet of self-discipline may also be reflected in the measure of frequency of religious service attendance, that is, those that have greater self-discipline might attend religious services more often. Future research should seek to examine the potential explanatory role of personality traits, and in particular, the facet of self-discipline, in the association between religiosity and problem gambling.

In terms of religious affiliation, overall, our findings are in line with previous research. In the current study, Catholic males had greater levels of problem gambling severity compared to Protestant males. A finding that is in line with previous research indicating that Catholics gambled more frequently and were more likely to be problem gamblers compared to Protestants [10, 11, 31, 32]. Furthermore, those with no religious affiliation (atheist/agnostic), tended to have lower levels of problem gambling severity compared to either Protestant or Catholic groups. This is similar to previous research indicating that Catholics were more likely to have gambled compared to those with no religious affiliation [11, 33]. As a potential explanation for the somewhat counterintuitive finding that religious affiliation can be associated with a greater probability of engaging in risky behaviors, findings from a recent study indicated that if a certain behavior is not prohibited by the ethical code of a given religious affiliation (which in the case of gambling is largely variant across denominations), transcendental beliefs might increase perceived control and thus risk taking [34].

In addition, those who preferred not to state their religious affiliation reported greater levels of problem gambling severity at baseline, compared to those who reported ‘Other’ religious affiliations. This may be because those in the ‘Other’ group were predominantly from religious traditions that either explicitly denounce gambling or deem it as culturally unacceptable (i.e., Islam, Buddhism, and Sikhism). In addition, it is also possible that those who preferred not to report their religious affiliation had less trust in the world and society, or had experienced trauma due to religion, both of which can be significant sources of stress and anxiety. Since gambling can be a maladaptive way of coping with negative emotions, it is possible that these potential heightened levels of stress and anxiety may have triggered an increase in gambling among this group. However, this is merely speculation, and due to the nature of self-reported data, it is unknown which category of religious affiliation (if any), these respondents who preferred not to state their religious affiliation, might identify with, nor the specific reasons underlying the decision to not report one’s religious affiliation.

Also of note is the independent influence of different measures of religion—religious affiliation, religious service attendance, and overall religiosity—on problem gambling severity among adults. These findings highlight the multi-dimensional nature of religion, and the importance of examining a variety of religion measures to accurately capture this construct [10, 27].

### Limitations

Given the low response rate (21.3%), the findings from the current study should be interpreted with caution. In addition, due to oversampling those at-risk, the QLS sample contained a somewhat higher proportion of At Risk, Problem, and Pathological Gamblers, and a lower proportion of Non-Gamblers compared to the province of Ontario, Canada (13.7% vs. 6.3%; 2.1% vs. 1.4%; 1.2% vs. 0.8%, respectively) [35]. However, according to the 2006 Canadian Census, the overall demographic profile of the QLS (i.e., gender, age, marital status, education, income, employment, and race/ethnicity) is similar to Canadian adults (15+), with some exceptions [16]. The QLS sample has a somewhat higher level of educational attainment, fewer people in the 18 to 24 and 65 years and older, age groups, as well as fewer single people, and visible minorities.

Although the religion measures used in the current study did influence the initial level of problem gambling severity, they did not influence the rate of change over time. One explanation for these null findings could be the use of time-invariant variables. It is likely that measures of religion are not static but instead change over time. Unfortunately, the QLS did not assess religiosity beyond the initial wave of data collection. Future research should seek to address this limitation using longitudinal datasets which capture measures of religiosity over time, and perhaps with the use of time-varying predictor variables and covariates, or alternatively with the use of a parallel-process growth curve model. Furthermore, the assumption in the current study, as well as all other studies conducted to date on this topic, is that religiosity *predicts* current and subsequent problem gambling severity. However, what has yet to be examined are potential causal effects in the opposite direction, and whether gambling predicts religiosity.

Another limitation revolves around the measure of religious affiliation. Specifically, with regards to the heterogeneity of the ‘Other’ category, which was comprised of diverse minority religions despite different perspectives on gambling. For example, within the Islamic tradition, gambling is explicitly prohibited in the Quran, whereas within the Jewish tradition, gambling is more culturally accepted and has a longstanding history. These diverse religions were grouped together because there was rather low variability in the population of Quinte overall; the ‘Other’ category represented only 9% of the total sample.

## Conclusions

The findings from the current study suggest that the relationship between religiosity and problem gambling is complex and nuanced. For example, the findings indicate that some dimensions of religiosity, including religious service attendance and personal religiosity may play a protective role against problem gambling, while other aspects of religiosity such as one’s religious affiliation were associated with an *increase* in problem gambling severity, such as the case with Catholics compared to Atheists. Thus, there is a need for additional research to better understand the relationship between problem gambling and religiosity over time taking into consideration additional factors that might cause individuals to select into organized religion, including but not limited to personality factors such as conscientiousness. Overall, the findings from this study provide support for the potential influential role of religious affiliation and respective faith-based doctrines in shaping cultural views on gambling and ultimately behavior.

## Additional files

Additional file 1: Table S1. Contrasts between all categories of religious affiliation when examining the influence of frequency of religious service attendance on the intercept and slope of problem gambling severity, by gender (*n* = 3959). Table presenting all contrasts between religious affiliation. (DOCX 14 kb)

Additional file 2: Table S2. Contrasts between all categories of religious affiliation when examining the influence of overall religiosity on the intercept and slope of problem gambling severity, by gender (*n* = 4120). Table presenting all contrasts between religious affiliation. (DOCX 14 kb)

## Abbreviations

CFI: Comparative Fit Index
FIML: Full Information Maximum Likelihood
PPGM: Problem and Pathological Gambling Measure
QLS: Quinte Longitudinal Study
RJRS: Rohrbaugh-Jessor Religiosity Scale
RMSEA: Root Mean Square Error of Approximation
SRMR: Standardized Root Mean Square Residual
TLI: Tucker-Lewis Index
