# Supplementary material for: The protective role of religiosity against problem gambling: findings from a five-year prospective study
Source: BMC Psychiatry. 2017 Nov 6;17:356. doi: 10.1186/s12888-017-1518-5 (PMC5674844; doi:10.1186/s12888-017-1518-5)
Supplement: Supplementary file 1 — Contrasts between all categories of religious affiliation when examining the influence of frequency of religious service attendance on the intercept and slope of problem gambling severity, by gender (n = 3959). Table presenting all contrasts between religious affiliation. (DOCX 14 kb) [file 12888_2017_1518_MOESM1_ESM.docx]

Table S1. Contrasts between all categories of religious affiliation when examining the influence of frequency of religious service attendance on the intercept and slope of problem gambling severity, by gender (n=3,959)

|  | Males (n=1,764) | | | | Females (n=2,195) | | | |
| --- | --- | --- | --- | --- | --- | --- | --- | --- |
|  | Intercept | | Slope | | Intercept | | Slope | |
|  | Effect | p | Effect | p | Effect | p | Effect | p |
| Protestant (ref.) |  |  |  |  |  |  |  |  |
| Catholic | 0.16 | 0.02 | 0.09 | 0.46 | 0.12 | 0.05 | -0.08 | 0.49 |
| Atheist/agnostic | -0.06 | 0.66 | 0.08 | 0.73 | -0.18 | 0.17 | 0.01 | 0.98 |
| Other | -0.16 | 0.09 | 0.10 | 0.54 | -0.31 | 0.001 | -0.003 | 0.98 |
| Prefer not to say | 0.16 | 0.18 | -0.30 | 0.15 | -0.02 | 0.85 | -0.12 | 0.50 |
| Catholic (ref.) |  |  |  |  |  |  |  |  |
| Protestant | -0.16 | 0.02 | -0.09 | 0.46 | -0.12 | 0.05 | 0.08 | 0.49 |
| Atheist/agnostic | -0.22 | 0.11 | -0.01 | 0.96 | -0.30 | 0.03 | 0.09 | 0.74 |
| Other | -0.32 | 0.002 | 0.01 | 0.95 | -0.43 | <0.001 | 0.07 | 0.68 |
| Prefer not to say | -0.01 | 0.96 | -0.39 | 0.08 | -0.14 | 0.18 | -0.04 | 0.83 |
| Other (ref.) |  |  |  |  |  |  |  |  |
| Catholic | 0.32 | 0.003 | -0.01 | 0.95 | 0.43 | <0.001 | -0.07 | 0.68 |
| Protestant | 0.16 | 0.09 | -0.10 | 0.54 | 0.31 | 0.001 | 0.003 | 0.98 |
| Atheist/agnostic | 0.10 | 0.50 | -0.02 | 0.93 | 0.13 | 0.41 | 0.01 | 0.97 |
| Prefer not to say | 0.31 | 0.02 | -0.40 | 0.10 | 0.29 | 0.02 | -0.12 | 0.61 |
