# Supplementary material for: The protective role of religiosity against problem gambling: findings from a five-year prospective study
Source: BMC Psychiatry. 2017 Nov 6;17:356. doi: 10.1186/s12888-017-1518-5 (PMC5674844; doi:10.1186/s12888-017-1518-5)
Supplement: Supplementary file 2 — Contrasts between all categories of religious affiliation when examining the influence of overall religiosity on the intercept and slope of problem gambling severity, by gender (n = 4120). Table presenting all contrasts between religious affiliation. (DOCX 14 kb) [file 12888_2017_1518_MOESM2_ESM.docx]

Table S2. Contrasts between all categories of religious affiliation when examining the influence of overall religiosity on the intercept and slope of problem gambling severity, by gender (n=4,120)

|  | Males (n=1,867) | | | | Females (n=2,253) | | | |
| --- | --- | --- | --- | --- | --- | --- | --- | --- |
|  | Intercept | | Slope | | Intercept | | Slope | |
|  | Effect | p | Effect | p | Effect | p | Effect | p |
| Protestant (ref.) |  |  |  |  |  |  |  |  |
| Catholic | 0.14 | 0.04 | 0.07 | 0.53 | 0.08 | 0.19 | -0.06 | 0.62 |
| Atheist/agnostic | -0.14 | 0.15 | 0.22 | 0.21 | -0.41 | <0.001 | 0.09 | 0.67 |
| Other | -0.19 | 0.04 | 0.11 | 0.47 | -0.36 | <0.001 | -0.01 | 0.97 |
| Prefer not to say | 0.09 | 0.44 | -0.24 | 0.26 | -0.02 | 0.81 | -0.16 | 0.39 |
| Catholic (ref.) |  |  |  |  |  |  |  |  |
| Protestant | -0.14 | 0.04 | -0.07 | 0.53 | -0.08 | 0.19 | 0.06 | 0.62 |
| Atheist/agnostic | -0.28 | 0.01 | 0.15 | 0.45 | -0.49 | <0.001 | 0.15 | 0.51 |
| Other | -0.33 | 0.001 | 0.04 | 0.82 | -0.44 | <0.001 | 0.05 | 0.78 |
| Prefer not to say | -0.05 | 0.70 | -0.31 | 0.16 | -0.10 | 0.32 | -0.10 | 0.62 |
| Other (ref.) |  |  |  |  |  |  |  |  |
| Catholic | 0.33 | 0.001 | -0.04 | 0.82 | 0.44 | <0.001 | -0.05 | 0.78 |
| Protestant | 0.19 | 0.04 | -0.11 | 0.47 | 0.36 | <0.001 | 0.01 | 0.97 |
| Atheist/agnostic | 0.05 | 0.71 | 0.11 | 0.63 | -0.05 | 0.71 | 0.09 | 0.71 |
| Prefer not to say | 0.28 | 0.04 | -0.35 | 0.15 | 0.33 | 0.01 | -0.15 | 0.51 |
